# The network and care coordination of ambulatory healthcare providers for people with mobility impairments: a cross-sectional network study involving stroke survivors and people with spinal cord injury in Germany

**DOI:** 10.1186/s12883-026-05208-6

**Published:** 2026-07-27

**Authors:** T. Schmittus, M. Wensing, N. Weidner, R. Rupp, J. Koetsenruijter

**Affiliations:** 1 Department of General Practice and Health Services Research, Heidelberg University Hospital, Heidelberg, Germany; 2 Spinal Cord Injury Centre, Heidelberg University Hospital, Heidelberg, Germany

**Keywords:** Network analysis, Inter-organizational, Care coordination, Rehabilitation, Primary care, Informal relationships, Natural network

## Abstract

**Background:**

People with mobility impairments such as stroke survivors or people with spinal cord injury (SCI) receive ambulatory healthcare from several healthcare providers. The coordination of these providers is challenging because there are usually no team meetings or shared treatment protocols. To gain insights into the coordinative processes of ambulatory care, the information transfer between providers was explored.

**Methods:**

A cross-sectional network study was conducted in an urban community in the south-west of Germany. First, an exploratory internet search for local providers was conducted. Then, providers were asked to distribute a survey to their patients (stroke, SCI) to identify the provider network. This was followed by a survey of the providers named by the surveyed patients. Network analysis was applied to explore connections and network characteristics (density, centrality).

**Results:**

Based on the patient survey (*n* = 22), a total of 58 healthcare providers in the community were identified, 20 of whom took part in the provider survey. In the network identified, physiotherapists, general practitioners, occupational therapists, orthopaedic technicians, neurologists, speech therapists and neuropsychologists were connected by 251 ties. The network had a density of 0.152 and a centrality of 0.442. Inter-organizational information sharing was only mentioned for 12 connections between healthcare providers. Care coordination in the community was rated as medium.

**Conclusion:**

Network analysis provided insight into a local network of healthcare providers. The network is dominated by a few providers and all providers have a low degree of connectivity. This study found that sharing of patient’s information between providers is rarely implemented.

**Supplementary Information:**

The online version contains supplementary material available at 10.1186/s12883-026-05208-6.

## Background

Stroke or Spinal Cord Injury (SCI) often leads to persistent motor impairments, activity limitations, and participation restrictions [1–4]. This requires rehabilitation with the aim to promote physical activity and healthy behaviour in order to maintain maximum functional capacity and avoid complications of disabling or progressive diseases [5]. The concept of rehabilitation is divided into different phases. These phases depend on the functional needs of the patient, e.g. whether it is an acquired or congenital condition and whether the condition is acute or has a progressive or degenerative course. Different phases of rehabilitation are described, from habilitation to rehabilitation in acute, post-acute and long-term settings [5]. It requires the provision of appropriate services and good planning of service delivery, capacity building and resourcing. Rehabilitation services should be stratified and organised in networks. The required resources include healthcare providers working in complex multi-professional teams [5]. After acute care and inpatient rehabilitation following a stroke or SCI, some patients require further rehabilitation services in the post-acute phase to further improve functioning, but also on the long term to maintain the obtained outcome [6–9]. In Germany, these post-acute rehabilitation services are provided on an ambulatory basis. Various healthcare providers are involved in the ambulatory rehabilitation and include, for example, general practitioners (GPs), neurologists, occupational therapists (OTs), physiotherapists (PTs), speech therapists, neuropsychologists, and orthopaedic technicians [9–11]. Outside Germany, physicians specialising in (physical and) rehabilitation medicine play a decisive role. The healthcare providers are supposed to collaborate in an inter-professional, coordinated, integrated, and patient-centred way to ensure high-quality care [8, 12–16]. In Germany, patients are free to choose their healthcare providers, with the exception that people with SCI are assigned a special medical supply store by their health insurance company. In Germany, the orthopaedic technicians work in medical supply stores and assistive products are available there. Some medical supply stores also have their own workshops where the assistive products are customised. There are ambulatory rehabilitation centres where several professional groups work for one employer [17]. In ambulatory medical centres, different healthcare providers are located in one building, but they work economically and organisationally separately from each other. Most ambulatory healthcare providers operate their practices independently and are spatially widespread [18]. The providers involved should therefore work together in an inter-organizational way to achieve coordinated care. This is typically done through the exchange of information between the different providers [19]. Several studies in Germany have shown that collaboration and information sharing between different healthcare providers is insufficient [16, 20–23]. Various reasons are assumed why inter-organizational care coordination does not occur, e.g., attitudes and habits of healthcare providers, lack of regulations, lack of time, and lack of reimbursement [24–26]. However, some providers probably collaborate voluntarily as a natural network that results from relationships among professionals through professional interests, referrals, support, friendships, communication, and consultation [27]. It is known that densely connected healthcare networks positively impact patient outcomes [28]. In a review, Stefano Tasselli [29] suggests a number of mechanisms that may explain the impact of healthcare networks. Some of these include: Organisational arrangements, such as rituals, teamwork, part-time work and case management, facilitate social interactions between professionals; Networks support the transfer of knowledge between different professions and promote multi-professional interaction. However, different professional groups tend to form cliques, with differences in their interaction patterns; Professional networks create barriers between different professional groups, that inhibit interprofessional interaction patterns and can delay the diffusion of innovations [29].

In the post-acute phase and in the long term, rehabilitation services in Germany are provided on an ambulatory basis. Team meetings, shared treatment protocols, and shared electronic patient files to coordinate healthcare delivery are not common among ambulatory care providers, although examples of those exist. Little is known about the extent to which these healthcare providers actually coordinate their treatment and care on the basis of information exchange on individual patients (e.g., written letters or telephone contacts). Only few studies have examined interorganizational connectivity between healthcare providers that are not integrated into an established network [30–32]. Considering that coordination of care can be challenging for ambulatory healthcare providers, deeper insights in the networks of information exchange connections could help to discover potential for improving care coordination. The aim of this study was to identify and explore connections between ambulatory healthcare providers in an urban community in Germany. We aimed to answer the following research questions: (1) Looking at shared patients, how is the network of local ambulatory healthcare providers for stroke survivors and people with SCI characterised? (2) To what extent do providers in this network share information on individual patients with each other?

## Methods

### Study design and setting

We conducted an observational, cross-sectional case study among patients and healthcare providers in one urban area. The area was a medium-large city (between 100,000 and 200,000 inhabitants), located in the southwest of Germany, and has an elaborated healthcare sector.

Social network analysis (SNA) is a research method for identifying and quantitatively analysing the connections between persons in a network and can be used to empirically describe, graph, and analyse the structure of the network [33, 34]. SNA is a proven method for identifying links between healthcare providers and their information flows [30, 35]. We first conducted a patient survey and subsequently conducted another survey among healthcare providers identified in the patient survey to document information exchange connections [34].

### Participants and recruitment

**Fig. 1:**
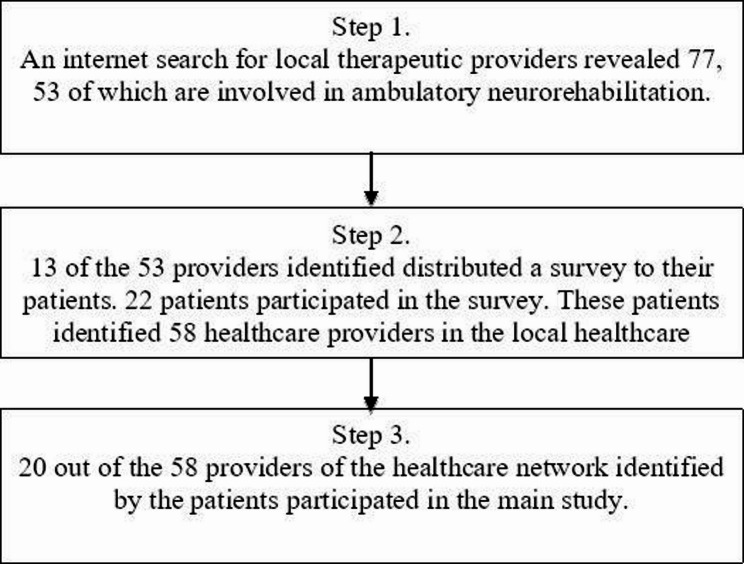
Recruitment flow chart

Study participants consisted of ambulatory healthcare providers treating patients after stroke or SCI. The selection of providers followed an iterative process (Fig. 1): Step (1) An explorative internet search of local providers in the field of ambulatory PT, OT, speech therapy, neuropsychology was performed. Providers’ websites revealed 77 providers (53 PT; 10 OT; 12 speech therapists; 2 neuropsychologists; 1 outpatient clinic for PT) potentially treating stroke and SCI patients. After contacting them, 53 were identified to be involved in healthcare activities for patients with stroke or SCI in the community. These providers were invited to participate in the study. Step (2) Participating providers were asked to distribute a written survey to their patients after stroke or SCI. This survey was used to find out which providers treated the same patient and thus establish a connection. We instructed the providers to pass the questionnaires on to the next 5 patients treated to achieve a random selection. The following inclusion criteria had to be met: current participation in therapy, diagnosis of stroke or SCI, impaired mobility, cognitively able to participate in the study, and at least 18 years old. Step (3) Finally, we conducted a written survey among the identified healthcare providers who had already been recruited after the initial internet search and additionally those who had been mentioned by patients in step 2, totalling 58 healthcare providers. The providers were invited to participate in the study by letter, fax or email and a reminder was sent out. One representative from each organization was surveyed, in most cases the owner or the specialized professional responsible for stroke or SCI.

### Survey questionnaires and data collection

Self-administered questionnaire surveys for patients and providers were conducted. The questionnaires were developed in German by the authors TS (health services researcher and practicing PT) and MW (health services researcher).

In the patient questionnaire, it was asked to name the healthcare providers visited in the last 12 months in a list (name generator). The relevant providers for the SNA were PTs, OTs, speech therapists, neuropsychologists, GPs, neurologists and orthopaedic technicians. Furthermore, patients’ characteristics (age, sex, stroke/SCI, years since the onset of the disease) were queried. Impairments were measured using the Barthel Index and participants were also asked to tick their symptoms such as pain, contractures, coordination and gait disorders in a list. Mobility status was assessed by asking whether the participant was a pedestrian, a person with mobility aids or a wheelchair user. The patient survey took place between October 2020 and June 2021 in paper and pencil form.

For the main part of the study, healthcare providers were surveyed to indicate with which practices or organizations they exchange information or have shared patients (see the provider questionnaire in Appendix 1). To collect data on the connections, a predefined list of healthcare providers (based on the patient survey) was used. The providers were invited to tick at each listed practice/organization whether they (a) have information exchange, (b) share patients, (c) know each other but have no information exchange and no shared patients, (d) know each other but do not know of any information exchange or shared patients or, (e) do not know each other. It was asked how information was exchanged (patient who passes on the information verbally /meetings/referrals/email/reports/fax/letter/telephone). In addition, we measured perceptions of the interorganizational care coordination between healthcare providers using a validated questionnaire by Brewster et al. [36]. The original questionnaire was used for measuring care coordination of elderly people and contains 12 items (questions) with response options consisted of a 5-point Likert scale ranging from 1 = strongly disagree to 5 = strongly agree. A forward and backward translation into German and adaptation of the wording to the target population of stroke and SCI was undertaken by TS and colleagues of the research institute. The last version was sent to the authors for review and was declared usable for this survey. Additional questions were asked to gain insight into meetings, positive experiences of care coordination and potential for improvement, provider socio-demographics, and organization-related questions. This survey took place between July and December 2021 and providers could choose a paper and pencil form or web-based platform named LimeSurvey via e-mailed web link.

Both questionnaires were pre-tested for comprehensibility with patients and providers who resembled the study participants. Appropriate adjustments were made, e.g. to individual terms and formatting.

### Data analysis

For SNA, we used the R software (version 4.2.1) with the user interface R studio (Version 2022.07.2, Build 576) and the igraph package (version 1.3.5). We graphically present the network in which we differentiate between connections based on shared patients and connections based on information exchange. A node represents a provider and lines between the nodes show who is connected to whom through information exchange (provider survey response (a) “have information exchange”) or only shared patients (patient survey and provider survey response (b) “shared patients”). In this study, at the network level, we calculated the indicators density (the degree to which providers were connected, theoretical range: 0 to 1) and centrality (the extent to which the network was dominated by one provider, theoretical range: 0 to 1), and, at the individual provider (ego) level, individual-level density (the degree to which each provider was connected, theoretical range: 0 to 1) [37–39].

SPSS (version 28.0.1.0) was used for descriptive statistics (frequencies and percentages) of participants characteristics and care coordination measurement. Free-text responses regarding the network and ways to improve care coordination were reviewed and thematically summarized.

## Results

### Study participants

The recruiting process is shown in Fig. 1 as a flow chart. Of the 77 providers contacted through the internet search, 53 were identified as being involved in the healthcare of patients with stroke or SCI in the community (Step1). For step 2, 13 providers agreed to carry out the patient survey. A total of 22 patients (recruited in 13 practices out of the 53 providers identified) participated in the survey, of which two questionnaires could not be used for the SNA because of missing values. From feedback of some providers, we learned that at that time, very few patients sought therapeutic treatment because they were afraid of contracting the Sars-CoV-2 virus.

The mean age of the 20 analysed patient data sets was 66.8 years, and they were affected for an average of 8.5 years (median 5). 16 persons were male and 16 were stroke survivors. Most symptoms were joint pain (*n* = 7), contractures and spasticity (*n* = 8), paralyses (*n* = 13), and coordination, balance or walking disorders (*n* = 15). The Barthel Index averaged 75.6 points. The mobility status was wheelchair user (*n* = 7), walker with an assistive products (*n* = 6), walker (*n* = 5), and walker and wheelchair user (*n*=2). In the last 12 months before the survey, all surveyed patients consulted PT and GP. Others were orthopaedic technicians (*n*=15), OT (*n*=16), neurologists (*n*=13), neuropsychotherapists (*n*=4), and speech therapists (*n*=3).

In step 2, a total of 58 healthcare providers were identified. 20 out of these 58 providers took part in the provider survey (step 3) (response rate was 34.5%). Rehabilitation professionals and orthopaedic technicians participated in the survey, but no GPs and neurologists could be recruited to participate. The characteristics of the 20 representatives of the participating healthcare providers can be found in Table 1. The average age was 48.6 years. Most of them were orthopaedic technicians or PTs and worked as owner/manager for the organization.

**Table 1:** Characteristics of the healthcare providers

	Total *n* (%) *N* = 20
Age	
Mean (SD; Min.-Max.) Missing values	48.6 (10.99; 31–62) 3 (15%)
Sex	
Male Female Missing values	9 (45%) 8 (40%) 3 (15%)
Profession	
Orthopaedic technician Physiotherapy Occupational therapy Neuropsychology Speech therapy Missing values	6 (30%) 5 (25%) 3 (15%) 2 (10%) 1 (5%) 3 (15%)
Employment	
Owner / manager Employed manager Employee Missing values	9 (45%) 6 (30%) 2 (10%) 3 (15%)
Patients with SCI treated/cared for in the last 12 months	
None Less than 10 10 to 20 20 to 50 More than 50 Missing values	2 (10%) 8 (40%) 2 (10%) 3 (15%) 2 (10%) 3 (15%)
Patients after stroke treated/cared for in the last 12 months	
None Less than 10 10 to 20 20 to 50 More than 50 Missing values	0 5 (25%) 3 (15%) 4 (20%) 5 (25%) 3 (15%)
Medical / rehabilitation professionals	
Mean (SD; Min.-Max.) Missing values	13.06 (14.86; 0–60) 4 (20%)
Specialisation for neurology	
Yes No Missing values	11 (55%) 5 (25%) 4 (20%)

### Network of ambulatory healthcare providers

A community-based network with a total of 58 ambulatory healthcare providers for people with stroke or SCI was identified. These healthcare providers covered PT *n* = 14, GP *n* = 13, OT *n* = 10, orthopaedic technology *n* = 10, neurology *n* = 6, speech therapy *n* = 3, and neuropsychology *n* = 2. These providers were connected by 251 ties through shared patients (the theoretical maximum was 1,653 ties). The number of average ties per provider was 8.66 (minimum 1; maximum 33). The network had a density of 0.152 and a centrality of 0.442. The eight providers with the highest density (d) were two orthopaedic technicians (RT2, d = 0.579; RT1, d = 0.491), a neurologist (NE1, d = 0.368), a physiotherapist (PT3, d = 0.351), an occupational therapist (OT7, d = 0.281), a speech therapist (ST4, d = 0.281), and two physiotherapists (PT14, d = 0.263; PT7, d = 0.246). The network characteristics of all providers can be found in Appendix 2.

**Fig. 2:**
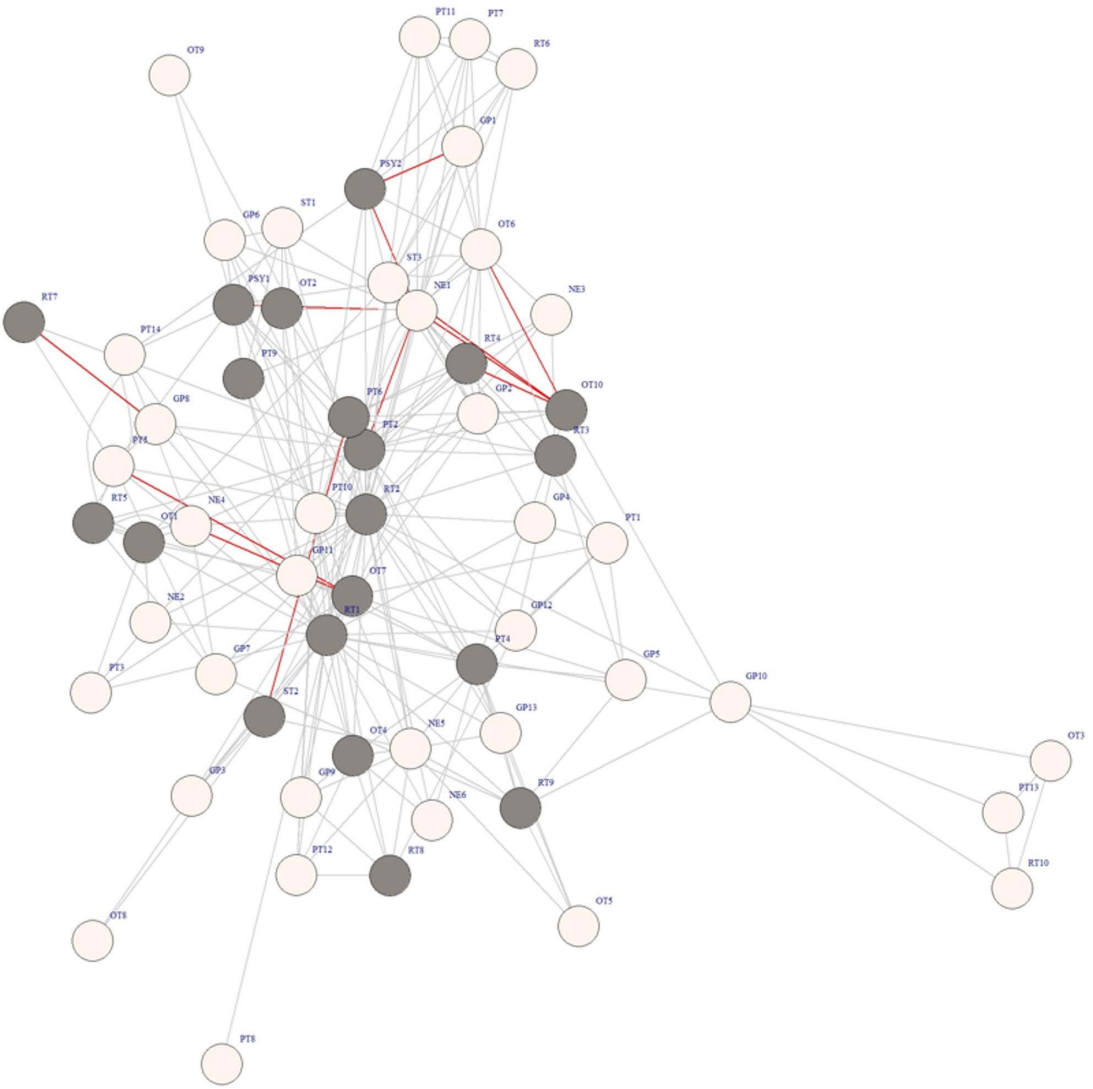
Network of ambulatory healthcare providers coloured by survey participation. Figure legend: grey nodes indicate survey participation (*n* = 20), white nodes no survey participation (*n* = 38); Grey lines indicate shared patients, red lines indicate information exchange (with or without shared patients); Abbreviations for professions: GP (general practitioner), NE (neurologist), PT (physiotherapist), OT (occupational therapist), ST (speech therapist), RT (orthopaedic technician), PSY (neuropsychotherapist)

Figure 2 (and Fig. 2.2 in Appendix 3) shows the identified network as a graph. Figure 2 illustrates participation in the survey and Fig. 2.2 (see Appendix 3) shows the network coloured by the professions of the providers. Connections between providers are shown with a grey line if two providers have one or more shared patients, and by a red line if they share information with each other. The graph illustrates that the network of healthcare providers is densely connected through shared patients. Also, the majority of providers forms one cluster and only one other cluster (3 white nodes at the bottom right of the Fig. 2) can be identified from the graph. This implies that most providers share patients with many others and not only with a few. On the other hand, the network based on information exchange (red lines) is much less dense, but with the constraint that these connections could only be indicated by the 20 providers included in the survey (grey nodes) who reported 12 connections that are based on exchange of information. The 20 providers could have mentioned a maximum of 950 information sharing connections, of which 1.3% was actually observed (density = 0.013).

Looking at the network positions of the different professional groups (see Appendix 3, Fig. 2.2), some physiotherapists and orthopaedic technicians are centrally located, and others are scattered in the outskirts. As the medical supply stores (orthopaedic technicians) are assigned to the patients with SCI, this could be the reason for their central position in the network. Other professional groups are distributed over the entire network.

### Care coordination in the community

The coordination of care in the community is assessed by the 20 providers who participated in the survey. The analysis of data based on the survey of Brewster et al. is shown in Table 2 [36]. On a scale with response options from 1 = strongly disagree to 5 = strongly agree, the mean score of the items was 2.9 (standard deviation (SD) 0.657) for alignment of healthcare strategy and 3.02 (SD 0.685) for coordination of current work. Up to 25% responded that they were involved in collaboration at least once a month.

**Table 2:** Care coordination measurement (adopted from Brewster et al. [36]; Only the scales are displayed)

Interorganizational collaboration scale (mean of items)	Mean (SD)^a^	Missing values
Factor 1—Aligning Strategy (6 items)^b^	2.90 (0.657)	8 (40%)
Factor 2—Coordinating Current Work (6 items)^c^	3.02 (0.685)	9 (45%)
Full scale (12 items)	2.92 (0.639)	9 (45%)

Collaboration behaviours - performed monthly or more	%	Missing values
Participation in coalition meetings	10%	15%
Communication about needs of individual clients	15%	15%
Reviews data on healthcare utilization together with other organizations that serve stroke / SCI patients in our community	25%	15%

^a^Response options consisted of a 5-point Likert scale with response options ranging from 1 = strongly disagree to 5 = strongly agree. Negatively worded items (R) were reverse-coded in the calculation of composite scores

^b^Questions #1, 2, 3, 4, 5, 12

^c^Questions #6, 7, 8, 9, 10, 11

Eight healthcare providers gave more detailed information about their meetings and named them such as a local stroke network, quality circle and regional group meeting for neuropsychologists, further training, patient-case meetings, a Parkinson network, co-operative meetings for orthopaedic technicians, and interdisciplinary therapy goal meeting. Six of these meetings mentioned were described as multi-professional. Open-ended responses regarding the network in the community and ways to improve care coordination were submitted by 11 providers. A few example quotes are given below:

“If all service providers involved have sufficient time for healthcare and an interprofessional exchange of information takes place, the quality of care is therefore above average.” (RT1).

“There are some medical services that cooperate really well and are easy to reach.” (OT10).

“If there was a network and a joint regulars’ table or something like this, that would be very positive.” (OT2).

In summary, providers said that they would like to see coordination of care, as coordination between organizations is necessary to achieve fast and good healthcare, but practically this only works in a few cases. It was said that care coordination does not work because there is a lack of time, interaction and information exchange about defined therapy goals and patient data. It was stated that knowing each other, initiating and participating in a network facilitates collaboration. The compilation of address lists of specialized therapists was suggested. Greater involvement in existing networks, more opportunities to coordination care, paid work time for the exchange of information and multi-professional trainings were called for.

## Discussion

This full network study of ambulatory healthcare providers for patients after stroke or SCI explored both patient sharing and information exchange. More providers were interconnected through shared patients than through the exchange of information about individual patients. This raises challenges for coordination of care, which were reflected in provider reported medium degree of care coordination. It is clear that many different providers are involved in a care network for stroke and SCI. However, the planning of service provision necessary for adequate rehabilitation is probably low due to the limited exchange of information, the necessary organisation in networks is also scarce and complex multi-professional teams are rarely implemented.

Although this explorative study provides new insight into coordination in community healthcare settings, some limitations of this study need to be mentioned. With the identified healthcare providers, we were probably not able to reach all potential patients, yet the maximum number of eligible patients was unknown. Some therapists reported that many patients stopped coming for treatments because of fear of Sars-CoV-2 contraction. Furthermore, it was not fully clear what the total number of healthcare providers for patients after stroke or with SCI was in the targeted community. Some providers may not consider themselves part of the healthcare network and did therefore not participate in the study [34, 40]. In addition, the participation of identified providers in the study was incomplete (34%) and selective as GPs and neurologists did not participate in the study. Connections identified may not be static but might fluctuate over time [29]. We only had data from one representative per healthcare provider, but the perception of relationships between providers can vary among employees within an organization. Due to the small study population and missing data from providers who did not participate, in-depth data analyses were not conducted. The generalizability of the results of the study is limited. Finally, there was a substantial number of missing values in the completed questionnaires. As a consequence of these limitations, the study may have underestimated the number of shared patients, information exchange, and related network coefficients such as density.

We identified the healthcare network at the level of the provider organisations (one professional in each organization). Recent research explored the interconnectivity of ambulatory healthcare providers at the patient level (ego-centred network) using SNA [41–44]. However, these studies mainly focused on professions rather than the individual providers in the community. Furthermore, the added value of a full network study is providing insights into connections between three or more actors. Other studies focused on purposefully established networks of health professionals, which are actually network organisations rather than naturally emerging networks [34, 45–49]. These networks are purposefully established, funded, or imposed by an agency, such as a government or organisation [27]. They may exist for a certain disease, e.g., Parkinson’s disease [45], or a certain profession, e.g., physicians [50, 51]. Further professional network studies were conducted within an institution, e.g., in a GP practice [33, 52–54], in a hospital [55–60], or on a hospital unit [61, 62]. However, this does not reflect ambulatory multi-professional and inter-organizational care coordination. Another area is the SNA-based analysis of secondary data, e.g., claims data from physician practices or hospitals [63, 64]. However, in this type of study, no data is available on whether providers exchange information.

In our study, the network centrality was 0.44 and density was 0.15. This means that the network is dominated by a few providers (centrality, theoretical range: 0 to 1) and all providers have a low degree of connectivity (density, theoretical range: 0 to 1). Other social network studies analysed multi-professional healthcare networks with 38 to 214 providers interconnected by 172 to 3,948 ties and a network density ranging from 0.04 to 0.60 [31, 34, 45–48]. The average number of ties per provider ranged from 8.9 to 17.4, and maximum numbers of ties per provider ranged from 28 to 41 [34, 45].

In our data, we found variability in professionals’ characteristics, such as the number of patients treated, which could have an impact on the position in the network. A study in the Netherlands found that professionals with more than 10 Parkinson’s disease patients had higher values for most network measures. Additionally, The geographic area and the healthcare setting (primary / hospital / both) had an impact on the measures [45]. Another study analysed mental health and social service networks in Brussels and London. Differences in the number of connections between the two cities were found for different types of services [31]. Lamontagne et al. [34] investigated a traumatic brain injury healthcare network in a province of Québec, Canada. It was found that relationships of members of an established consortium were denser than between others. A Canadian study by Gainforth et al. [46, 47] examined interpersonal communication in the implementation of physical activity guidelines for people with SCI among staff in a community-based organization. It was found that interpersonal communication was greater within the network’s centre than between the centre and periphery and within the periphery. Factors that impact the central positions were greater knowledge of evidence-based resources and greater engagement in promoting physical activity. In a study by Groenen et al. [48], SNA was used to identify the profession for the case manager role in maternity care in a single region in the Netherlands. Obstetricians and community-based midwives were found to be the most central, with community-based midwives making 51% of all ties and increasing the network density by 25%, but each profession had central individuals in the network.

With our results, we were not clear which health profession plays a central role in ambulatory care for stroke and SCI survivors, because the highest density measures varied among different professions, especially for orthopaedic technicians and PTs. We were not able to show in our data that GPs play the central role in connecting involved providers, as another study suggests [16]. There is a lack of specialists in (physical and) rehabilitation medicine in Germany, which could lead to coordination and networking not being as well established as it should be. The role of specialists and GPs is to work together to prescribe and coordinate rehabilitation care to ensure more regular follow-up care. In the targeted domain of healthcare, the role of GPs as boundary spanners in supporting inter-organizational collaboration seems to be challenging [65]. A network model of rehabilitation services across the phases and stages of care is essential and could overcome the current challenges.

In our study, it was shown that a small proportion of the shared patient connections are professional contacts with information exchange between providers. The study by Wensing et al. [45] showed that 46% of connections consisted of professional contacts. Participation in coalition meetings was reported by 10% of our 20 participating providers. In another study, about one-third of ties were routine meetings [31]. In our study, coordination of care was rated medium although there were very few communicative connections between the 20 participating providers. This is probably because the providers did not know the overall network [34]. Another reason could be that professionals do not perceive other professions as part of the healthcare team and care coordination varies between different professions [40]. In the studies by Barzel et al. [15, 23] the cooperation of therapists with GPs was rated as dissatisfying, while GPs were satisfied with the cooperation with therapists. Our findings show that while there are efforts at interprofessional networking, this does not reach all providers in the community or that providers express interest for networking but do not actively seek it out. Collegial meetings were held, some of which were interprofessional. Sharing information was found fundamental for good quality of care by the study participants. Coordination of care can be achieved in different, sometimes complementary ways, e.g., through shared guidelines/protocols even without further communication, collegial meetings (often in the hospital setting), written information about individual patients (common for the ambulatory setting) or patients managing their care themselves. It is expected that some patients will establish exchanges of information between different healthcare providers involved in patient care (possibly without the providers’ knowledge of this), such as patient-held notes, which can be supplied in hard copy, or on a loaned electronic device. Our results suggest that it seems to be advantageous for the ambulatory providers to actively communicate with each other by telephone or in meetings. One way to improve communication between providers could be electronic patient files. A positive effect of this electronic data access is the provision of patient data to providers who have access to it. This could lead to improved information sharing [66, 67]. There are also indications that communication tools integrated into the electronic health record can improve the exchange of information [68]. Nevertheless, there are also indications that a personal exchange of information is necessary in complex patient cases [69]. The study by Hunt et al. [70] provides indications that the role of a coordinator and multidisciplinary team meetings are effective for improving collaboration and coordination of healthcare for people with Severe Mental Illness.

Despite the limitations of the study, the data provide insights into outpatient healthcare and provide care-relevant data that can be seen as preliminary work for further studies. Even though a lot of data is missing in this study, the results show a trend: Care coordination may not run optimally because there is a lack of information exchange by providers. Presumably, a defined service provider (e.g. a rehabilitation physician) is missing as an active boundary spanner in this non-official network. In future studies, it would be useful to determine the number of users of healthcare services (patients), which leads to the identification of the entire network of healthcare providers. This information could help to investigate unknown networks.

## Conclusions

Through applied network analysis, we were able to map the healthcare network of ambulatory healthcare providers in an urban community. Identifying relevant healthcare providers in not established networks can be challenging if there are no general registrations. Assuming that there are mostly no formal networks in ambulatory care in Germany, it seems reasonable in future studies to develop strategies to strengthen networking of healthcare providers. This examination should include investigating the extent to which this improves patient outcomes and influences other healthcare data. Service providers are inevitably linked to each other through shared patients. Being aware of this and proactively promoting the exchange of information could help to establish networking structures and achieve better coordinated care. Politicians should create structures and a financing model that enable networking. Digitalization (e.g. electronic patient files) could make a significant contribution to this.

## Supplementary Information

Supplementary Material 1. Appendix 1: Survey instrument.

Supplementary Material 2. Appendix 2: Network characteristics of the individual healthcare providers.

Supplementary Material 3. Appendix 3: Figure 2.2. Network of ambulatory healthcare providers coloured by the profession.

## Data Availability

No datasets were generated or analysed during the current study.

## Abbreviations

GGP: General practitioner
OT: Occupational therapist
PT: Physiotherapist
SCI: Spinal cord injury
SD: Standard deviation
SNA: Social network analysis

## References

[1] Feigin VL, Brainin M, Norrving B, et al. World Stroke Organization (WSO): Global Stroke Fact Sheet 2022. Int J Stroke. 2022;17(1):18–29. 34986727 10.1177/17474930211065917

[2] Kumar R, Lim J, Mekary RA, et al. Traumatic Spinal Injury: Global Epidemiology and Worldwide Volume. World Neurosurg. 2018;113:345–63. 10.1016/j.wneu.2018.02.033 29454115

[3] van den Berg MEL, Castellote JM, Mahillo-Fernandez I, et al. Incidence of Spinal Cord Injury Worldwide: A Systematic Review. Neuroepidemiology. 2010;34(3):184–92. 20130419 10.1159/000279335

[4] Strøm V, Månum G, Arora M, et al. Physical Health Conditions in Persons with Spinal Cord Injury Across 21 Countries Worldwide. J Rehabil Med. 2022;54:302. 10.2340/jrm.v54.2040 PMC9272839 35678293

[5] European Physical and Rehabilitation Medicine Bodies Alliance. White Book on Physical and Rehabilitation Medicine (PRM) in Europe. Chapter 8. The PRM speciality in the healthcare system and society. Eur J Phys Rehabil Med. 2018;54(2):261–78. 10.23736/S1973-9087.18.05152-3 29565109

[6] Thorsén AM, Holmqvist LW, de Pedro-Cuesta J, et al. A randomized controlled trial of early supported discharge and continued rehabilitation at home after stroke: five-year follow-up of patient outcome. Stroke. 2005;36(2):297–303. 15618441 10.1161/01.STR.0000152288.42701.a6

[7] Düchs C, Schupp W, Schmidt R, et al. Schlaganfallpatienten nach stationärer neurologischer Rehabilitation der Phase B und C: Durchführung von Heilmittelbehandlungen und Arztkontakte in einem Langzeitverlauf von 2,5 Jahren nach Entlassung [Stroke Patients after Neurological In-patient Rehabilitation Phases B and C: Application of Therapeutic Measures and Contact to General Practitioners during an Aftercare Period of 2.5 Years]. Phys Med Rehab Kuror. 2012;22(3):125–33.

[8] Gemperli A, Ronca E, Scheel-Sailer A, et al. Health care utilization in persons with spinal cord injury: part 1 - outpatient services. Spinal Cord. 2017;55(9):823–7. 28462932 10.1038/sc.2017.44

[9] Bychkovska O, Tederko P, Engkasan JP, et al. Healthcare service utilization patterns and patient experience in persons with spinal cord injury: a comparison across 22 countries. BMC Health Serv Res. 2022;22(1):755. 35672727 10.1186/s12913-022-07844-3 PMC9175375

[10] National Clinical Guideline Centre (UK). Rehabilitation after traumatic injury NG211. 2022. Available from: https://www.nice.org.uk/guidance/ng211. Cited 2022 Dec 14.

[11] National Clinical Guideline Centre (UK). Stroke Rehabilitation: Long Term Rehabilitation After Stroke. 2013. Available from: https://www.ncbi.nlm.nih.gov/books/NBK247494/pdf/Bookshelf_NBK247494.pdf.

[12] Langhorne P, Bernhardt J, Kwakkel G. Stroke rehabilitation. Lancet. 2011;377(9778):1693–702. 21571152 10.1016/S0140-6736(11)60325-5

[13] Leijten FRM, Struckmann V, van Ginneken E et al. The SELFIE framework for integrated care for multi-morbidity: Development and description. Health Policy. 2018;122(1):12–22. 10.1016/j.healthpol.2017.06.002 28668222

[14] Powell Davies G, Williams AM, Larsen K, et al. Coordinating primary health care: an analysis of the outcomes of a systematic review. Med J Aust. 2008;188(S8):65–8. 10.5694/j.1326-5377.2008.tb01748.x 18429740

[15] Barzel A, Eisele M, van den Bussche H. Die ambulante Versorgung von Schlaganfallpatienten aus Sicht von Hamburger Hausärzten - eine explorative Studie [Outpatient Management of Stroke Patients from the Viewpoint of General Practitioners in Hamburg - An Exploratory Study]. Gesundheitswesen. 2008;70(03):170–6. 18415925 10.1055/s-2008-1062731

[16] Nolte CH, Jungehülsing GJ, Rossnagel K, et al. Schlaganfallnachsorge wird von Hausärzten erbracht. Kooperative Strategien für Patienten mit chronischen Defiziten nach Schlaganfall sind nicht die Regel [Stroke aftercare is provided by GPs. Cooperative strategies for patients with chronic deficits after stroke are not the standard]. Nervenheilkunde. 2009;28(03):135–7.

[17] Pöppl D, Deck R, Kringler W, et al. Strukturen und Prozesse in der ambulanten Neurorehabilitation [Structures and Processes in Outpatient Neurorehabilitation]. Rehabilitation (Stuttg). 2014;53(03):168–75. 24399283 10.1055/s-0033-1353193

[18] Nolte E, Knai C, Hofmarcher M, et al. Overcoming fragmentation in health care: chronic care in Austria, Germany and The Netherlands. Health Econ Policy Law. 2012;7(1):125–46. 22221931 10.1017/S1744133111000338

[19] McDonald KM, Sundaram V, Bravata DM et al. Vol. 7: Care Coordination. In: Shojania K, McDonald K, Wachter R, editors. Closing the Quality Gap: A Critical Analysis of Quality Improvement Strategies. Technical Review 9 (Prepared by the Stanford University-UCSF Evidence-based Practice Center under contract 290-02-0017). Vol. AHRQ Publication No. 04(07)-0051-7. Rockville (MD): Agency for Healthcare Research and Quality; 2007.

[20] Barzel A, Ketels G, Schön G, et al. Erste deutschlandweite Befragung von Physio- und Ergotherapeuten zur Berufssituation. Teil 3: Physio- und Ergotherapeuten zwischen Kooperation und Konkurrenz [First Germany-wide Survey of Physiotherapists and Occupational Therapists on the Professional Situation. Part 3: Physiotherapists and Occupational Therapists between Cooperation and Competition]. physioscience. 2011;7(3):91–8.

[21] Hempler I, Maun A, Kampling H, et al. Schlaganfallnachsorge in Deutschland. Ergebnisse einer Online-Befragung stationär und ambulant tätiger Experten in Süddeutschland [Poststroke care in Germany. Results of an online survey of inpatient and outpatient experts in southern Germany]. Nervenarzt. 2019;90(8):824–31. 30617567 10.1007/s00115-018-0655-5

[22] Hempler I, Woitha K, Thielhorn U, et al. Post-stroke care after medical rehabilitation in Germany: a systematic literature review of the current provision of stroke patients. BMC Health Serv Res. 2018;18(1):468. 29914476 10.1186/s12913-018-3235-2 PMC6006784

[23] Barzel A, Eisele M, van den Bussche H. Ambulante Versorgung von Schlaganfallpatienten aus der Sicht Hamburger Physio- und Ergotherapeuten [Ambulatory Care of Stroke Patients from the View of Physiotherapists and Occupational Therapists in Hamburg]. physioscience. 2007;3(4):161–6.

[24] Supper I, Catala O, Lustman M, et al. Interprofessional collaboration in primary health care: a review of facilitators and barriers perceived by involved actors. J Public Health (Oxf). 2015;37(4):716–27. 25525194 10.1093/pubmed/fdu102

[25] Sangaleti C, Schveitzer MC, Peduzzi M, et al. Experiences and shared meaning of teamwork and interprofessional collaboration among health care professionals in primary health care settings: a systematic review. JBI Database Syst Rev Implement Rep. 2017;15(11):2723–88. 10.11124/JBISRIR-2016-003016 29135752

[26] Daniel T, Spingler T, Hug A, et al. Current practice of outpatient rehabilitation services in patients with mobility-impaired paralysis due to stroke or spinal cord injury: a qualitative interview study in Germany. Disabil Rehabil. 2024;46(17):3922–36. 10.1080/09638288.2023.2259301 37732606

[27] Braithwaite J, Runciman WB, Merry AF. Towards safer, better healthcare: harnessing the natural properties of complex sociotechnical systems. Qual Saf Health Care. 2009;18(1):37–41. 19204130 10.1136/qshc.2007.023317 PMC2629006

[28] Ostovari M, Yu D. Impact of care provider network characteristics on patient outcomes: Usage of social network analysis and a multi-scale community detection. PLoS ONE. 2019;14(9):e0222016. 31498827 10.1371/journal.pone.0222016 PMC6733513

[29] Tasselli S. Social Networks of Professionals in Health Care Organizations:A Review. Med Care Res Rev. 2014;71(6):619–60. 25380607 10.1177/1077558714557079

[30] Chambers D, Wilson P, Thompson C, et al. Social Network Analysis in Healthcare Settings: A Systematic Scoping Review. PLoS ONE. 2012;7(8):e41911. 22870261 10.1371/journal.pone.0041911 PMC3411695

[31] Nicaise P, Tulloch S, Dubois V, et al. Using social network analysis for assessing mental health and social services inter-organisational collaboration: findings in deprived areas in Brussels and London. Adm Policy Ment Health. 2013;40(4):331–9. 22543978 10.1007/s10488-012-0423-y

[32] Dearing JW, Beacom AM, Chamberlain SA, et al. Pathways for best practice diffusion: the structure of informal relationships in Canada’s long-term care sector. Implement Sci. 2017;12(1):11. 28159009 10.1186/s13012-017-0542-7 PMC5291985

[33] Scott J, Tallia A, Crosson JC, et al. Social network analysis as an analytic tool for interaction patterns in primary care practices. Ann Fam Med. 2005;3(5):443–8. 10.1370/afm.344 PMC1466914 16189061

[34] Lamontagne ME. Exploration of the integration of care for persons with a traumatic brain injury using social network analysis methodology. Int J Integr Care. 2013;13:38. 10.5334/ijic.1055 PMC3821538 24250281

[35] Goodwin N. It’s good to talk: social network analysis as a method for judging the strength of integrated care. Int J Integr Care. 2010;10:e120. 21289998 10.5334/ijic.647 PMC3031854

[36] Brewster AL, Tan AX, Yuan CT. Development and application of a survey instrument to measure collaboration among health care and social services organizations. Health Serv Res. 2019;54(6):1246–54. 31595498 10.1111/1475-6773.13206 PMC6863239

[37] Carrington PJ, Scott J. The SAGE Handbook of Social Network Analysis. London: Sage; 2011. Available from: http://digital.casalini.it/9781446250112.

[38] Wasserman S, Faust K. Social network analysis: methods and applications. Cambridge: Cambridge University Press Cambridge; 1994.

[39] Provan K, Sebastian J. Networks within networks: Service link overlap, organizational cliques, and network effectiveness. Acad Manage J. 1998;08/01:41:453–63.

[40] Doekhie KD, Buljac-Samardzic M, Strating MM, et al. Who is on the primary care team? Professionals’ perceptions of the conceptualization of teams and the underlying factors: a mixed-methods study. BMC fam prac. 2017;18(1):111. 10.1186/s12875-017-0685-2 PMC5745958 29281980

[41] Weenink J-W, van Lieshout J, Jung HP, et al. Patient Care Teams in treatment of diabetes and chronic heart failure in primary care: an observational networks study. Implement Sci. 2011;2011/07(03):66. 10.1186/1748-5908-6-66 PMC3143081 21722399

[42] Fernández-Peña R, Molina JL, Valero O. Personal Network Analysis in the Study of Social Support: The Case of Chronic Pain. Int J Environ Res Public Health. 2018;15(12):2695. 30501074 10.3390/ijerph15122695 PMC6313565

[43] Grol SM, Molleman GRM, Wensing M, et al. Professional Care Networks of Frail Older People: An Explorative Survey Study from the Patient Perspective. Int J Integr Care. 2020;20(1):12. 32292310 10.5334/ijic.4721 PMC7147679

[44] Crotty MM, Henderson J, Ward PR, et al. Analysis of social networks supporting the self-management of type 2 diabetes for people with mental illness. BMC Health Serv Res. 2015;15:257. 26138825 10.1186/s12913-015-0897-x PMC4490681

[45] Wensing M, van der Eijk M, Koetsenruijter J, et al. Connectedness of healthcare professionals involved in the treatment of patients with Parkinson’s disease: a social networks study. Implement Sci. 2011;2011/07(03):67. 10.1186/1748-5908-6-67 PMC3150321 21722400

[46] Gainforth HL, Latimer-Cheung AE, Athanasopoulos P, et al. The role of interpersonal communication in the process of knowledge mobilization within a community-based organization: a network analysis. Implement Sci. 2014;9(1):59. 2014/05/22. 24886429 10.1186/1748-5908-9-59 PMC4052292

[47] Gainforth HL, Latimer-Cheung AE, Moore S, et al. Using Network Analysis to Understand Knowledge Mobilization in a Community-based Organization. Int J Behav Med. 2015;22(3):292–300. 25187110 10.1007/s12529-014-9430-6 PMC4449387

[48] Groenen CJM, van Duijnhoven NTL, Faber MJ et al. Use of social network analysis in maternity care to identify the profession most suited for case manager role. Midwifery. 2017;45:50–5. 10.1016/j.midw.2016.12.007 28024229

[49] Schoenfelder J, Zarrin M, Griesbaum R, et al. Stroke care networks and the impact on quality of care. Health Care Manag Sci. 2022;25(1):24–41. 34564805 10.1007/s10729-021-09582-0 PMC8983551

[50] Landon BE, Keating NL, Onnela JP, et al. Patient-Sharing Networks of Physicians and Health Care Utilization and Spending Among Medicare Beneficiaries. JAMA Intern Med. 2018;178(1):66–73. 29181504 10.1001/jamainternmed.2017.5034 PMC5833496

[51] Paul S, Keating NL, Landon BE, et al. Reprint of: Results from using a new dyadic-dependence model to analyze sociocentric physician networks. Soc Sci Med. 2015;125:51–9. 25442972 10.1016/j.socscimed.2014.08.027

[52] Wensing M, van Lieshout J, Koetsenruiter J, et al. Information exchange networks for chronic illness care in primary care practices: an observational study. Implement Sci. 2010;5(1):3. 2010/01/22. 20205758 10.1186/1748-5908-5-3 PMC2822738

[53] Holtrop JS, Ruland S, Diaz S, et al. Using Social Network Analysis to Examine the Effect of Care Management Structure on Chronic Disease Management Communication Within Primary Care. J Gen Intern Med. 2018;33(5):612–20. 2018/05/01. 29313225 10.1007/s11606-017-4247-z PMC5910335

[54] Heijmans N, van Lieshout J, Wensing M. Information exchange networks of health care providers and evidence-based cardiovascular risk management: an observational study. Implement Sci. 2017;12(1):7. 10.1186/s13012-016-0532-1 PMC5237141 28086813

[55] Uddin S, Hossain L, Hamra J, et al. A study of physician collaborations through social network and exponential random graph. BMC Health Serv Res. 2013;2013/06/26(1):234. 10.1186/1472-6963-13-234 PMC3695881 23803165

[56] Mascia D, Cicchetti A, Damiani G. Us and Them: a social network analysis of physicians’ professional networks and their attitudes towards EBM. BMC Health Serv Res. 2013;13(1):429. 2013/10/22. 24148207 10.1186/1472-6963-13-429 PMC3815661

[57] Carson MB, Scholtens DM, Frailey CN, et al. Characterizing Teamwork in Cardiovascular Care Outcomes: A Network Analytics Approach. Circ Cardiovasc Qual Outcomes. 2016;9(6):670–8. 28051772 10.1161/CIRCOUTCOMES.116.003041 PMC5217475

[58] Curtis DE, Hlady CS, Kanade G, et al. Healthcare worker contact networks and the prevention of hospital-acquired infections. PLoS ONE. 2013;8(12):e79906. 24386075 10.1371/journal.pone.0079906 PMC3875421

[59] Mundt MP, Swedlund MP. A human factors systems approach to understanding team-based primary care: a qualitative analysis. Fam Pract. 2016;33(6):721–6. 27578837 10.1093/fampra/cmw093 PMC5161491

[60] Mundt MP, Gilchrist VJ, Fleming MF, et al. Effects of primary care team social networks on quality of care and costs for patients with cardiovascular disease. Ann Fam Med. 2015;13(2):139–48. 25755035 10.1370/afm.1754 PMC4369607

[61] McCurdie T, Sanderson P, Aitken LM. Applying social network analysis to the examination of interruptions in healthcare. Appl Ergon. 2018;67:50–60. 10.1016/j.apergo.2017.08.014 29122200

[62] van Beek AP, Wagner C, Spreeuwenberg PP, et al. Communication, advice exchange and job satisfaction of nursing staff: a social network analyses of 35 long-term care units. BMC Health Serv Res. 2011;11:140. 21631936 10.1186/1472-6963-11-140 PMC3133544

[63] Brunson JC, Laubenbacher RC. Applications of network analysis to routinely collected health care data: a systematic review. J Am Med Inf Assoc. 2018;25(2):210–21. 10.1093/jamia/ocx052 PMC6664849 29025116

[64] Arnold C, Koetsenruijter J, Forstner J, et al. Influence of physician networks on prescribing a new ingredient combination in heart failure: a longitudinal claim data-based study. Implement Sci. 2021;16(1):84. 2021/08/28. 34454547 10.1186/s13012-021-01150-y PMC8401102

[65] Kousgaard MB, Joensen AS, Thorsen T. The challenges of boundary spanners in supporting inter-organizational collaboration in primary care - a qualitative study of general practitioners in a new role. BMC Fam Pract. 2015;16:17. 25887910 10.1186/s12875-015-0231-z PMC4355472

[66] Vos JFJ, Boonstra A, Kooistra A, Seelen M, van Offenbeek M. The influence of electronic patient file record use on collaboration among medical specialties. BMC Health Serv Res. 2020;20(1):676. 32698807 10.1186/s12913-020-05542-6 PMC7374868

[67] Menachemi N, Collum TH. Benefits and drawbacks of electronic health record systems. Risk Manag Healthc Policy. 2011;4:47–55. 22312227 10.2147/RMHP.S12985 PMC3270933

[68] Gandhi TK, Keating NL, Ditmore M, Kiernan D, Johnson R, Burdick E, Hamann C. Improving Referral Communication Using a Referral Tool Within an Electronic Medical Record. In: Henriksen K, Battles JB, Keyes MA, Grady ML, editors. Advances in Patient Safety: New Directions and Alternative Approaches. Performance and Tools. Volume 3. Rockville (MD): Agency for Healthcare Research and Quality (US); 2008 Aug. 21249931

[69] Sheu L, Fung K, Mourad M, Ranji S, Wu E. We need to talk: Primary care provider communication at discharge in the era of a shared electronic medical record. J Hosp Med. 2015;10(5):307–10. 25755159 10.1002/jhm.2336

[70] Hunt CM, Spence M, McBride A. The role of boundary spanners in delivering collaborative care: a process evaluation. BMC Fam Pract. 2016;17:96. 27473529 10.1186/s12875-016-0501-4 PMC4966858

