# Supplementary material for: The network and care coordination of ambulatory healthcare providers for people with mobility impairments: a cross-sectional network study involving stroke survivors and people with spinal cord injury in Germany
Source: BMC Neurol. 2026 Jul 27;26:479. doi: 10.1186/s12883-026-05208-6 (PMC13404645; doi:10.1186/s12883-026-05208-6)
Supplement: Supplementary file 1 — Supplementary Material 1. Appendix 1: Survey instrument. [file 12883_2026_5208_MOESM1_ESM.pdf]

Appendix 1: Survey instrument

NOTE: This is an anonymized version of the survey instrument. Study participants completed a web-based version or paper and pen version of this survey that was tailored to their community and listed specific facilities.

Questionnaire for healthcare providers that serve stroke and spinal cord injury patients in [name of city]

Dear study participant,

Thank you for taking the time to answer this questionnaire. Ambulatory healthcare for mobility-impaired patients involves a variety of healthcare providers and services, which presents a challenge for care coordination and communication between the various providers. With this questionnaire, we would like to learn about your experiences regarding communication between your facility and other facilities in [name of the city] **for patients with stroke or spinal cord injury**.

The questionnaire will take about 25 minutes to complete (depending on how many facilities your facility has contact with). You can decide not to answer questions at any time. However, complete answers to all questions are crucial for the evaluation. The information is evaluated pseudonymously (encrypted) and is only accessible to the researchers involved in the study.

The questionnaire is divided into several parts:

Part 1: Information exchange and shared patients	In a (very long – but that should not deter you, as it is quick to coplete) table, you will be asked about your exchange of information or shared patients with other facilities in the city.
Part 2: Your information exchange with five of the facilities from Part 1	For the facilities for which you indicated "yes" in Part 1, we ask for more detailed information on how you exchange information with five of them.
Part 3: Assessment of the care coordination between health and social services	We would like to know your overall impression of the care coordination between health and social services in the city.
Part 4: Your impression of the current care coordination	Let us know what you think about the care coordination.
Part 5: Key data about you and your facility	This information is important for interpreting the results.

If possible, answer the questions from the perspective of your entire facility (including colleagues), not just your personal one.

Please return the completed questionnaire to us within two weeks. If you have any questions, please contact: [RESEARCHER CONTACT DETAILS].

Please note the following information when filling out the questionnaire:

- Use ballpoint pen only.
- Please enter free text information in block letters.
- Tick the boxes clearly: X
- Please do not tick between the boxes.
- If you accidental make an incorrect selection, please fill in the box completely and tick your selection again: ■ X
- There are no wrong and right answers.
- Please fill out the questionnaire as completely as possible.

Please return the completed questionnaire to us in the prepaid return envelope.

Thank you for your participation!

Part 1: Information exchange and shared patients

Below you will find a list of some practices and medical supply stores in the city. **Information exchange** means that your facility is in contact with the other. This can be an exchange of information about shared patients or other care-related topics. **Shared patients** are patients who are cared for or treated by your facility AND by other facilities, even if you are not aware of this. If your facility has an exchange of information with another or at least one shared patient, **please complete an attachment sheet (part 2)**. Here we ask you to provide information on the 5 most important facilities for the care of mobility-impaired patients with stroke or spinal cord injury.

1.1. Do you know these **PHYSIOTHERAPY** practices? (Please tick the box that best applies.)					
	Yes, we have exchanged information in the last 12 months. **Please fill out an attachment sheet in Part 2 in this regard.**	Yes, we have shared stroke/spinal cord injury patients, but no exchange of information.	I know that this practice exists. But we have no exchange of information and shared patients.	I know that this practice exists. But I don't know if we have exchanged information and shared patients.	No, this practice is unknown to me.
No.1 [Name of practice]	☐	☐	☐	☐	☐
No.2 …	☐	☐	☐	☐	☐
…	☐	☐	☐	☐	☐

NOTE: The above table is repeated for each healthcare profession.

Part 2: Your information exchange with five of the facilities from questionnaire part 1

In this part of the questionnaire, we are interested in the exact process of your information exchange with other facilities. For the facilities for which you ticked "Yes, we have exchanged information in the last 12 months." (first table column) in Part 1, we now ask for more detailed information on information exchange. Please select 5 of these facilities. If you would like to complete more, further forms are available.

Part 2: Appendix 1 for further information on information exchange [only one template is presented here]

1. Number of the facility (transfer from the list) for which you wish to provide further information: ______________
2. How many patients from your facility are also treated or cared for by this practice/medical supply store (shared patients)? (e.g., through prescriptions / referrals / recommendations) (please specify number)

☐ Total: _____ Of which stroke patients (approximately): _____ Of which paraplegics (approximately): _____

☐ I don't know

☐ None

3. What content do you exchange with each other? (tick all boxes that apply)

☐ Information about patients (e.g. examination results, therapy reports)

☐ Coordination of care (e.g. extended therapies, further examinations, etc.) in patient cases

☐ Common goal setting regarding therapies, assistive devices, etc. for patients

☐ Organizational matters relating to patient cases (e.g., changes to the frequency of therapy)

☐ Exchange of information on medical-therapeutic or care-related topics (e.g., new treatment approaches, changes in the catalogue of therapeutic products, aspects of care in [name of the city])

☐ None

☐ Other: ____

4. How detailed is the information you receive when you need it? (only tick one box)

☐ Very good: All/additional information is shared.

☐ Good: The important/necessary information is shared.

☐ Mediocre: Partially missing information.

☐ Insufficient: Necessary information cannot be obtained.					
5. How often does your facility exchange information with the other practice/medical supply store? (only tick one box)					
☐ Never ☐Daily ☐Weekly ☐Monthly ☐5 to 10 times per year ☐Less than 5 times per year					
6. How often does your facility use the following information channels?					
	Never	Rare	Sometimes	Often	Always
It is the patient who passes on the information verbally.	☐	☐	☐	☐	☐
We exchange information via meetings (face-to-face meeting / video conference).	☐	☐	☐	☐	☐
We exchange information by prescription/referral.	☐	☐	☐	☐	☐
We exchange information by e-mail.	☐	☐	☐	☐	☐
We exchange information by fax.	☐	☐	☐	☐	☐
We exchange information by therapy report/doctor's letter.	☐	☐	☐	☐	☐
We exchange information by letter.	☐	☐	☐	☐	☐
We exchange information by phone.	☐	☐	☐	☐	☐
We exchange information during random meetings (e.g., in the stairwell/elevator).	☐	☐	☐	☐	☐
We exchange information (additionally) privately.	☐	☐	☐	☐	☐
Other (please specify):	☐	☐	☐	☐	☐
7. How often does the information exchange take place in a timely manner?	☐	☐	☐	☐	☐
8. How often are problems in patient care solved together?	☐	☐	☐	☐	☐

In the following parts of the questionnaire, we would like to know your overall impression and opinion of the care coordination between all health care facilities and social services in [name of the city]. We need information about you and your facility to interpret the results.

Part 3: Your assessment of the care coordination between health and social services

NOTE: Here follows the survey questionnaire by Brewster et al.* Part C. C-1.
* Brewster AL, Tan AX, Yuan CT. Development and application of a survey instrument to measure collaboration among health care and social services organizations. Health Services Research. 2019;54(6):1246-1254.

If possible, please give us some information about one of the above-mentioned meetings (e.g., doctors' network, quality circle, therapists' regulars' table):
Name / type of meeting:<br>Approximately how many people attend these meetings:<br>Which specialist professions are represented:<br>From which facilities (e.g., acute/rehabilitation clinic, GPs/specialists, remedial practices etc.):<br>Meeting place / location:

NOTE: Here follows the survey questionnaire by Brewster et al.* Part C. C-2.
* Brewster AL, Tan AX, Yuan CT. Development and application of a survey instrument to measure collaboration among health care and social services organizations. Health Services Research. 2019;54(6):1246-1254.

Part 4: Your impression of the care coordination

1. Please tell us POSITIVE things about the care coordination with other facilities in [name of the city]:

2. Please let us know whats need to be improved in the care coordination with other facilities in [name of the city]:

Part 5: Key data about your facility

1. What position are you currently in? (tick only 1 box)

☐ Owner/Managing director

☐ Employed with management function

☐ Employed

☐ Freelancer

☐ Other:______________________________________

2. Which employment situation applies to you? Employment is understood to mean any paid activity. (tick only 1 box)

☐ Full-time employment (38.5 hours per week or more)

☐ Part-time employment (less than 38.5 hours per week)

3. Which professional group do you belong to? (tick only 1 box)

☐ Ggeneral practitioner	☐ Occupational therapist
☐ Neurologist	☐ Speech therapist
☐ Psychologist	☐ Rehabilitation / Orthopedic technician
☐ Neuropsychologist	☐ Other:____
☐ Physiotherapist	

4. How many years of professional experience (after graduation) do you have **in the ambulatory healthcare sector**?

Please specify number: __________ years

5. What specializations/certificates in **neurology** do you have **at your facility**? Please name the most important ones in bullet points:

6. How long has your facility existed (year of foundation)?

Please indicate the year: |___|___|___|___|

7. Does your facility have a license from public health insurances?

☐ Yes ☐ No

8. Do you use electronic data management (practice software or similar) in your facility?

☐ Yes ☐ No

9. How many medical / therapeutic professionals (e.g., doctors, therapists, orthopaedic technicians) work in your facility? (Number of people)
Please specify number: ______________
10. How many other employees (e.g., medical assistants, administration, patient management) work in your facility? (Number of people)
Please specify number: ____________
11. Does your facility specialize in neurology?
☐ Yes ☐ No

12. What is the percentage of neurological patients in your facility?											
☐ <5%	☐ 5%	☐ 10%	☐ 20%	☐ 30%	☐ 40%	☐ 50%	☐ 60%	☐ 70%	☐ 80%	☐ 90%	☐ 100%

13. How many **stroke** patients have been treated/cared for in your facility in the last 12 months?
☐ Zero ☐ less than 10 ☐ between 10 and 20 ☐between 20 and 50 ☐ more than 50
14. How many patients with **spinal cord injury** have been treated/cared for in your facility in the last 12 months?
☐ Zero ☐ less than 10 ☐ between 10 and 20 ☐between 20 and 50 ☐ more than 50

15. Your year of birth?	16. Your sex?
|___|___|___|___|	☐ Male ☐ Female ☐ Diverse

You have made it!! Thank you for your participation!

Please return the completed questionnaire to us in the prepaid return envelope.

If you have any questions, please contact: [RESEARCHER CONTACT DETAILS].
