# Supplementary material for: The network and care coordination of ambulatory healthcare providers for people with mobility impairments: a cross-sectional network study involving stroke survivors and people with spinal cord injury in Germany
Source: BMC Neurol. 2026 Jul 27;26:479. doi: 10.1186/s12883-026-05208-6 (PMC13404645; doi:10.1186/s12883-026-05208-6)
Supplement: Supplementary file 2 — Supplementary Material 2. Appendix 2: Network characteristics of the individual healthcare providers. [file 12883_2026_5208_MOESM2_ESM.pdf]

Appendix 2: Network characteristics of the individual healthcare providers

ID	EGO DEGREE (NO. OF TIES)	DENSITY (DEGREE / 58-1)
**RT2**	33	0,579
**RT1**	28	0,491
**NE1**	21	0,368
**PT3**	20	0,351
**OT7**	16	0,281
**ST4**	16	0,281
**PT14**	15	0,263
**PT7**	14	0,246
**PSY1**	13	0,228
**PT10**	13	0,228
**OT10**	12	0,211
**NE5**	12	0,211
**GP13**	11	0,193
**OT5**	10	0,175
**NE4**	10	0,175
**PSY2**	10	0,175
**RT4**	10	0,175
**OT13**	9	0,158
**RT3**	9	0,158
**RT5**	9	0,158
**OT2**	8	0,140
**GP1**	8	0,140
**GP2**	8	0,140
**PT9**	8	0,140
**PT13**	8	0,140
**RT9**	8	0,140
**OT1**	7	0,123
**GP7**	7	0,123
**GP10**	7	0,123
**GP11**	7	0,123
**GP15**	7	0,123
**ST1**	7	0,123
**PT2**	7	0,123
**PT11**	7	0,123
**PT15**	7	0,123
**RT6**	7	0,123
**GP8**	6	0,105
**GP14**	6	0,105
**PT17**	6	0,105
**PT21**	6	0,105
**RT8**	6	0,105
**GP3**	5	0,088
**GP5**	5	0,088
**GP9**	5	0,088
**ST3**	5	0,088

Table continued

ID	EGO DEGREE (NO. OF TIES)	DENSITY (DEGREE / 58-1)
**NE2**	5	0,088
**NE3**	5	0,088
**OT6**	4	0,070
**GP4**	4	0,070
**NE6**	4	0,070
**PT4**	4	0,070
**OT3**	3	0,053
**PT19**	3	0,053
**RT7**	3	0,053
**RT10**	3	0,053
**OT11**	2	0,035
**OT12**	2	0,035
**PT12**	1	0,018
