# Supplementary material for: The network and care coordination of ambulatory healthcare providers for people with mobility impairments: a cross-sectional network study involving stroke survivors and people with spinal cord injury in Germany
Source: BMC Neurol. 2026 Jul 27;26:479. doi: 10.1186/s12883-026-05208-6 (PMC13404645; doi:10.1186/s12883-026-05208-6)
Supplement: Supplementary file 3 — Supplementary Material 3. Appendix 3: Figure 2.2. Network of ambulatory healthcare providers coloured by the profession. [file 12883_2026_5208_MOESM3_ESM.pdf]

Appendix 3: Figure 2.2. Network of ambulatory healthcare providers coloured by the profession

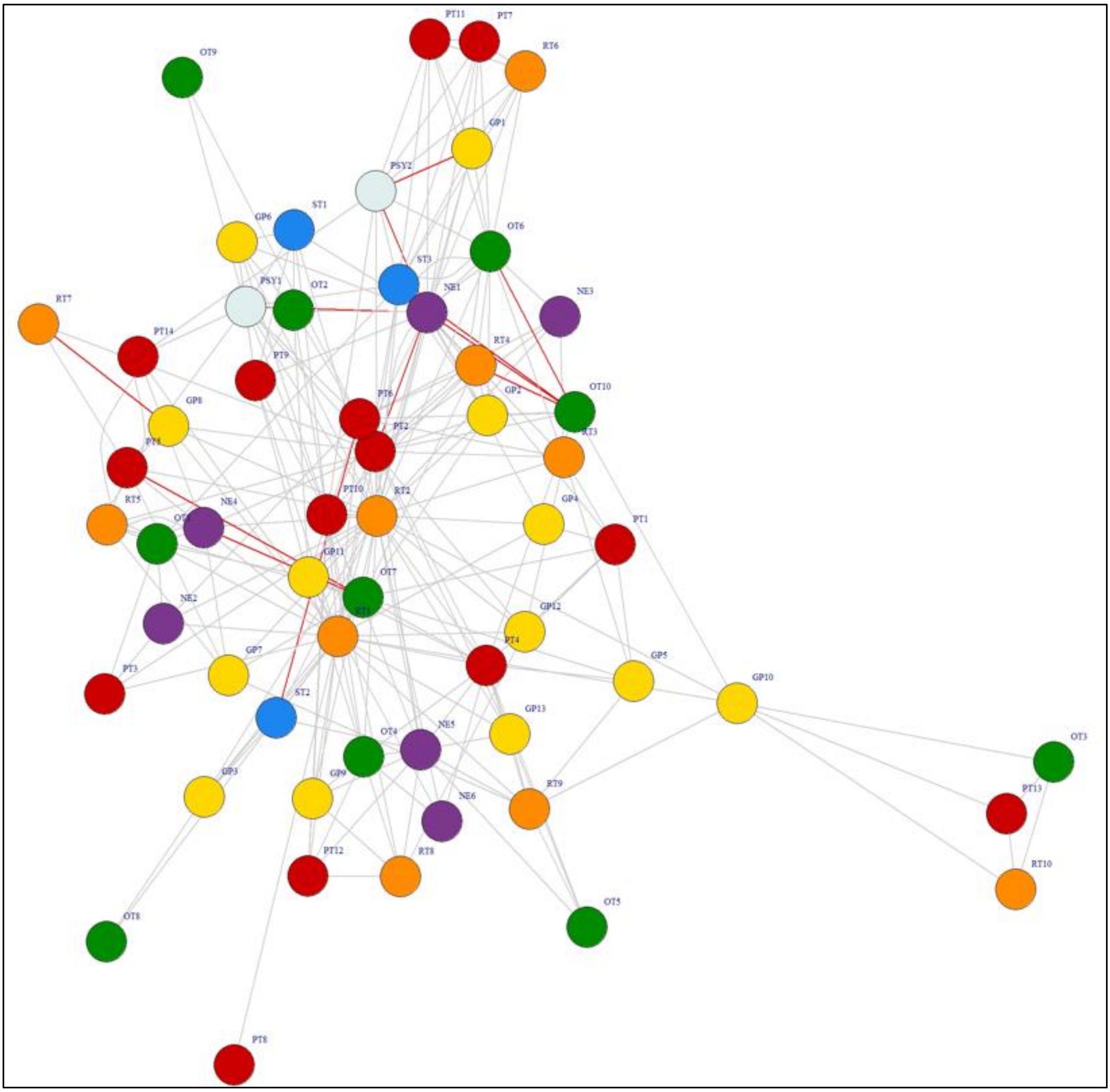

Fig. 2.2. Network of ambulatory healthcare providers coloured by profession. Figure legend: Grey lines indicate shared patients, red lines indicate information exchange; Abbreviations and colours for professions: GP (general practitioner, yellow), NE (neurologist, purple), PT (physiotherapist, red), OT (occupational therapist, green), ST (speech therapist, blue), RT (rehabilitation technician, orange), PSY (psychotherapist, light grey).
